# The New Journal, Hopes and Aims for the Future

**Published:** 1990-03

**Authors:** 


### THE NEW JOURNAL, HOPES AND AIMS FOR THE FUTURE

After a year of overtures and discussion the new alliance has been formed, with the two West of England faculties of the Royal College of General Practitioners and most of the Specialist Clubs joining the Bristol Medico-Chirurgical Society and the Clinical Society of Bath. A local journal has become a regional journal, we have moved up a division in the league tables. What should be our aims?

**'To produce a journal that our readership of West Country doctors will open with anticipation and read with satisfaction'.** 'Easier said than done, a euphoric platitude, you'll never please everybody, try again'. Well, since 1986 when the *Bristol Medico-Chirurgical Journal* began to be distributed throughout the West Country it has developed the West Country connection with local correspondents. Now with its new affiliations the *West of England Medical Journal* will have more friends and be better placed to attract good material from the whole of the west.

**It will be a general medical journal** and this is a threatened species. Increasingly medical journals are devoted to a specialist interest, of necessity they become more technical and unreadable except by the initiated. It is important for us all to read articles on subjects outside our own clinical interest, written by specialists for the general reader. Such an article becomes especially readable (and assessable) if it is written by someone you know personally. The West of England has become a clinical entity. With its web of specialist clubs and its faculties of general practice we all know each other as we have never done before and we can write for each other. The specialist club abstracts are a valuable archive, they are useful when trying to recall details of papers previously heard. It is also valuable to glance through abstracts of alien specialities and find out what they are up to. We have our correspondents who will tell us about the things of importance in their localities. We would like to encourage every West country doctor who has special knowledge, or a special hate, or a bee in his bonnet to write about it, either as a letter, an annotation or a paper. Local medical history is of great interest and importance and can only be recorded in columns such as ours. Every profession has a different slant on life and doctors are no exception, we have things to say about life that are worth saying, even if they are not strictly about our practice and we have our own brand of humour and our hobbies.

**And then there are the burning issues**, just now the potential disaster about to overwhelm the NHS or the wind of change that will bring in all the benefits of healthy competition—which view is right? It is no use reading the White Paper which contains only vague intentions and pious platitudes. The debate must go on in the open, in the regions, for all to enter. The government 'presses on regardless' with its panacea, the B.M.A's initial blunderbus opposition lost it many friends outside the profession but inside few support the proposals for general practice. The impact on the hospital scene of the '*Enthoven Internal Market*' is much more difficult to assess, but the more it is thought through the more counterproductive it appears likely to be. A coherent consensus has yet to emerge. We invite particularly people who have views on the hospital scene in our region to write for us.

The new journal is the product of an affiliation of West of England medical groups but without sponsorship it would not have been born. Nuffield Hospitals have not only provided the sponsorship but also encouraged the old journal to grow into the new one. We have a chance to be relevant to the West country scene. **There are aspects of medicine that can be served by a regional journal that other journals cannot cover**.

